# Mesenchymal stem cell carriers enhance antitumor efficacy of oncolytic adenoviruses in an immunocompetent mouse model

**DOI:** 10.18632/oncotarget.17557

**Published:** 2017-05-02

**Authors:** Esther Rincón, Teresa Cejalvo, Deepak Kanojia, Arantzazu Alfranca, Miguel Ángel Rodríguez-Milla, Raul Andrés Gil Hoyos, Yu Han, Lingjiao Zhang, Ramón Alemany, Maciej S. Lesniak, Javier García-Castro

**Affiliations:** ^1^ Unidad de Biotecnología Celular, Instituto de Salud Carlos III, Madrid, Spain; ^2^ The Brain Tumor Center, The University of Chicago, Chicago, Illinois, USA; ^3^ Institut Català d´Oncologia, IDIBELL, Barcelona, Spain

**Keywords:** mesenchymal stem cells, carriers, oncolytic adenoviruses, immunotherapy, cancer

## Abstract

Oncolytic virotherapy represents a promising alternative for cancer treatment; however, viral delivery to the tumor represents a major challenge. Mesenchymal stem cells (MSCs) chemotax to tumors, and can serve as a viral delivery tool. Previously, we demonstrated antitumor therapeutic efficacy for mesenchymal stem cells (MSCs) infected with the oncolytic human adenovirus ICOVIR5 (Celyvir) for treatment of neuroblastoma patients. Given the lack of suitable immunocompetent preclinical models, the mechanism underlying Celyvir antitumor activity remains unknown. In this study, we used the syngeneic murine CMT64 cell line as a human adenovirus-semi-permissive tumor model and demonstrate the homing capacity of mouse Celyvir (mCelyvir) to CMT64 tumors. We found that the combined treatment of mCelyvir and intratumoral injections (i.t.) of ICOVIR5 was more effective than treatment with i.t. ICOVIR5 alone. Interestingly, the superior therapeutic effect of the combined therapy was associated with a higher tumor infiltration of CD8+ and CD4+ T cells. Our findings suggest that the use of MSCs as carriers of oncolytic adenovirus can improve the clinical efficacy of anti-cancer virotherapy, not only by driving the adenovirus to tumors, but also through their potential to recruit T cells.

## INTRODUCTION

A correlation between the level of immune cell infiltration and the clinical outcome has been detected in many cancers [1]. While antitumor immunotherapy has primarily focused on activating T cells and inhibiting immune checkpoints [2], oncolytic viruses may represent an alternative strategy for cytoreduction of tumors. Not only do oncolytic viruses combat tumors via direct lysis and/or vascular attack, but they also potently activate the adaptive and innate immune responses [3]. In fact, oncolytic therapy is generally thought as an immunotherapy and the term ‘oncolytic immunotherapy’ has been widely adopted [4].

Among oncolytic viruses used in clinical trials, oncolytic adenoviruses (Ad) present several unique characteristics, including relatively simple genetic modification for tumor selectivity, and high viral titer production [5]. Unfortunately, the efficacy of Ad-induced tumor reduction has been limited in clinical trials due to insufficient viral delivery to the tumor, and because the virus triggers the immune response that attenuates viral production [6]. Mesenchymal stem cells (MSCs) have the intrinsic capability to migrate to solid tumor and have been employed to deliver anti-cancer agents, including oncolytic Ad, specifically to the tumor. This method reduces systemic side effects, and promotes effective local anti-tumor activity. [7–9]. In addition, the ability of MSCs to modulate the immune system has been suggested to be crucial for their benefits in antitumor strategies [10–13].

Our groups have been using MSCs as delivery vehicles to improve oncolytic Ad delivery. We have previously demonstrated their potential benefits in human glioma xenografts in immunodeficient mice [14, 15], in a semi-permissive cotton rat model [10], and in the treatment of patients with metastatic neuroblastoma [12, 16]. In humans, patients who responded to this therapy had significantly higher counts of absolute numbers and a different kinetics of circulating T lymphocytes during treatment, suggesting a main role of immune system in clinical responses [12, 16]. However, a major limitation in the field is the lack of immune- and replication-competent models that are necessary to elucidate the mechanisms underpinning MSC-delivered, oncolytic Ad antitumor therapy. Given this limitation, in this study we have used a semi-permissive mouse model to study key immune system components that can be modulated to improve the therapy, and thereby improve the survival of patients with metastatic tumors. The development of an immunocompetent model has been hampered by the fact that most mouse cells do not support the replication of human Ad [17, 18]. However, murine lung carcinoma cell lines CMT64 and KLN205 support the complete replication cycle of human Ad [19–21]. In this regard, the potential benefits of using MSCs as a carrier for oncolytic Ad in these models have not been evaluated.

The present study aimed to understand the immune mechanisms involved in the antitumor therapeutic efficacy of the treatment with oncolytic Ad delivered using MSCs as vehicles. We have used the semi-permissive CMT64 cell line, syngeneic for C57BL/6 mice, as a replication-competent model to analyze the efficacy of murine MSCs (mMSCs) infected with the human oncolytic Ad ICOVIR5 (mCelyvir) [22, 23], as cell carriers of this Ad. ICOVIR5 replicated and induced cytopathic effect in CMT64 cells. Combined *in vivo* treatment with mCelyvir and ICOVIR5 intratumoral injections (i.t.) shrank tumors by 50%, a higher decrease than tumors treated only with ICOVIR5 (i.t.). Interestingly, the superior therapeutic effect of combined mCelyvir and ICOVIR5 was associated with a higher tumor infiltration of CD8+ and CD4+ T lymphocytes, suggesting a main role of immune system in efficacy of Celyvir.

## RESULTS

### Replication and cytotoxicity of ICOVIR5 in mouse CMT64 cells and mMSCs

**Figure 1:**
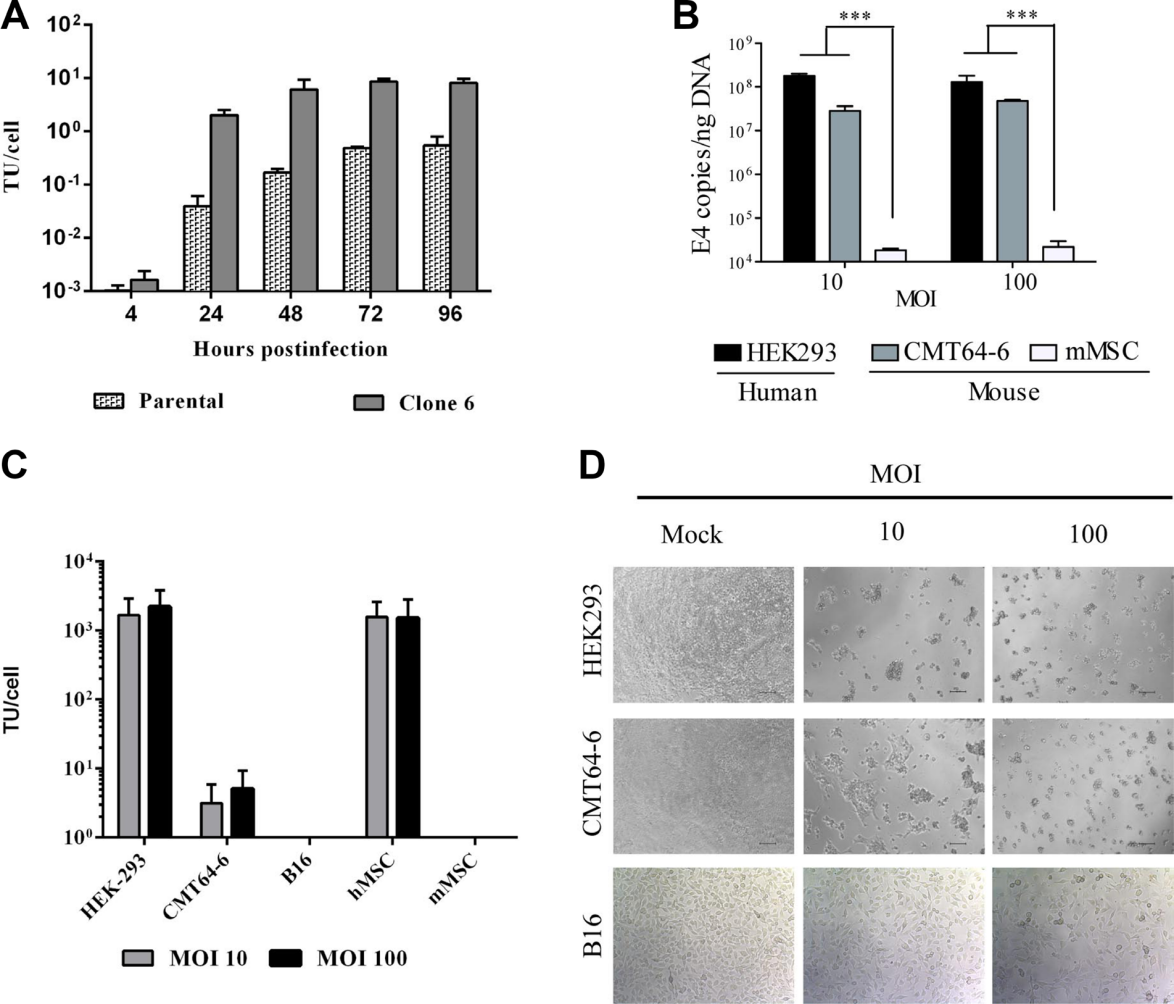
The murine CMT64-6 cell line supports replication of the human adenovirus ICOVIR5 (**A**) CMT64 parental cells and CMT64-6 clone cells were infected with human oncolytic adenovirus at MOI 200 during 4 hours. Cellular extracts were obtained at 4, 24, 48, 72 and 96 h and the amount of virus produced was determined by hexonprotein staining using the Adeno-X Rapid Titer Kit protocol. Bars represent mean ± SEM. (**B**) Quantitative PCR detection of viral replication. HEK293, CMT64-6 cells and mMSCs were infected as previously and, 3 days post-infection, the degree of viral genome amplification was assessed by measuring the number of viral E4A copy/ng DNA. Bars represent mean of triplicates ± SEM. ANOVA was performed and statistical significance was defined as ***P < 0.001. (**C**) HEK-293, CMT64-6, B16, hMSC and mMSC viral production ability was evaluated. Cells were infected with ICOVIR5 at 10 and 100 MOI and, after 72 h, cellular extracts were obtained to determine virus production as in Figure 1a. (**D**) HEK293 cells (as human permissive cells, positive control), CMT64-6 cells, and B16 cells (as non-permissive murine cells, negative control) were infected with different MOIs (10 and 100 infection unit/cell) and cytopathic effect was evaluated after 72 h. Each experiment was performed at least 3 times.

The murine non-small-cell lung carcinoma cell line CMT64 had been described to be semi-permissive to human Ad infection [19]. This data led us to hypothesize that some cells were producing virus while other cells were not, possibly due to heterogeneity in Ad life cycle. To avoid possible heterogeneity problems in virus production by the parental CMT64 cells, we isolated 30 different CMT64 clones by single cell isolation in 96 well plates and measured virus production in these clones (Supplementary Figure 1A). Among them, clone 6 (CMT64-6) was selected as it produced 5 to 10 TU/cell (Figure 1A, Supplementary Figure 1A). The higher production of Ad in CMT64-6 compared to the parental CMT64 cells could be caused by a better infectivity of this clone. To test this possibility, CMT64 parental and clone 6 were infected with AdTL, an E1- and E3-deleted recombinant serotype 5 Ad that contains a green fluorescence protein (EGFP) and luciferase gene-expression cassette [24, 25]. The percentage of transduced cells was analyzed by fluorescence microscopy. The results indicated that when infected at an MOI of 50 TU/cell, almost 100% of the cells of clone 6 were transduced compared to 20 % infection of the parental cells (Supplementary Figure 1B).

**Figure 2:**
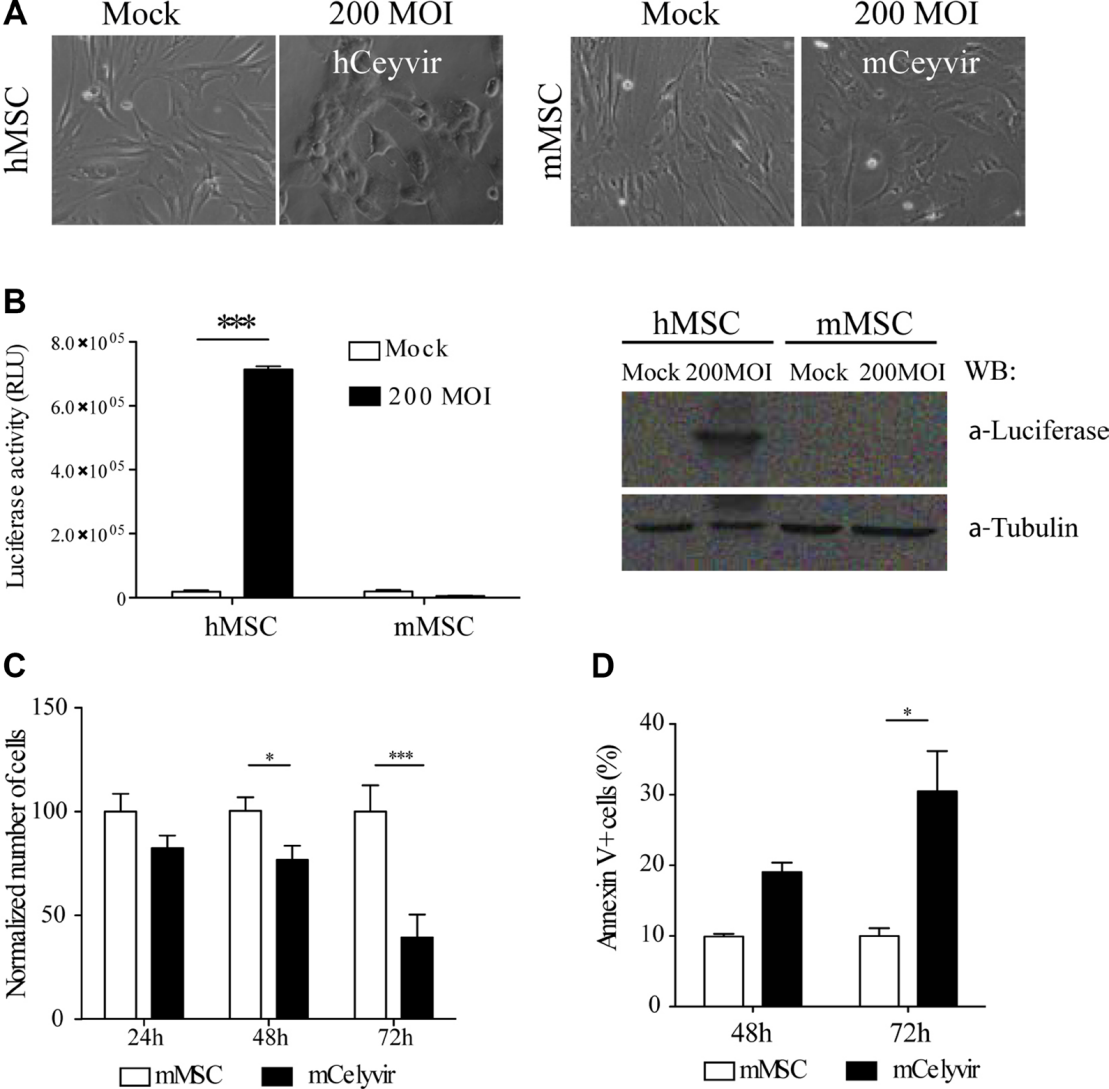
Permissiveness of mesenchymal stem cells to ICOVIR5 (**A**) hMSCs and mMSCs were infected with ICOVIR15Luc Ad at 200 MOI and the morphology of both cell types was evaluated after 48 h. (**B**) The pellets of the cells previously evaluated were lysated and luciferase activity was measured, as a readout of Ad replication. The protein expression of luciferase was also analyzed by western blot in those lysates. (**C**) mMSCs viability was compared to mCelyvir viability (mMSCs infected with 200 infection unit/cell of ICOVIR5) by trypan blue exclusion method, at the indicated time points after infection. The number of cells is shown as fold change compared to 100% for the non-infected cells. (**D**) Apoptosis cell death associated with ICOVIR5 infection was evaluated in mMSCs, 48 h and 72 h after infection. The percentage of apoptotic Annexin V+ analyzed by FACS is represented. Bars represent mean of triplicates ± SEM. Student's *t* test was performed and statistical significance was defined as *P < 0.05, **P < 0.01 and ***P < 0.001.

We therefore decided to use CMT64-6 for further experiments. To measure viral replication, adenoviral E4A gene copies were quantified 72 h after ICOVIR5 infection (Figure 1B). The level of ICOVIR5 replication in CMT64-6 cells in this assay was approximately 5-fold lower than that detected in human HEK293 cells, a highly permissive human cell line of reference. We evaluated ICOVIR5 viral production in CMT64-6 cells compared to other cell lines, and observed that this cell line produces some ICOVIR5, but non-permissive cells, like the murine melanoma cell line B16 or mMSCs, failed to produce any Ad (Figure 1C). ICOVIR5 production yield in CMT64-6 cells was lower than the one observed in highly permissive cells, as HEK293 and human MSCs (Figure 1C). A clear cytopathic effect in culture was also detected on CMT64-6 cells, similar to that observed in human HEK293 cells. That effect was not observed in B16 cells (Figure 1D). These results indicate that CMT64-6 cells represent a mouse tumor model semi-permissive to human Ad ICOVIR5 replication. By contrast, mMSCs obtained from adipose tissue [26] (Supplementary Figure 2), were not able to amplify the ICOVIR5 genome (Figure 1B, 1C), even though the transduction efficiency of these cells infected with AdTL was higher than 90% (Supplementary Figure 1C). Accordingly, we did not observe cytopathic effects in mMSC compared to human MSC (Figure 2A). These results were further confirmed using ICOVIR15Luc (a human ICOVIR5 derivative, expressing the Firefly-luciferase as an additional splicing unit of the major late promoter), which allows correlation of luciferase activity with Ad replication. As expected, luciferase activity was detected only in ICOVIR15Luc-infected human MSCs. The same results were obtained by western blot using an anti-Luciferase antibody (Figure 2B). While mMSCs were not replicating virus, their population was still reduced after Ad infection (Figure 2C) due to apoptosis (Figure 2D). These findings indicate that, although human Ad does not replicate in mMSCs, it is still toxic for these cells.

### CMT64-6 cell tumors induce homing of mCelyvir

**Figure 3:**
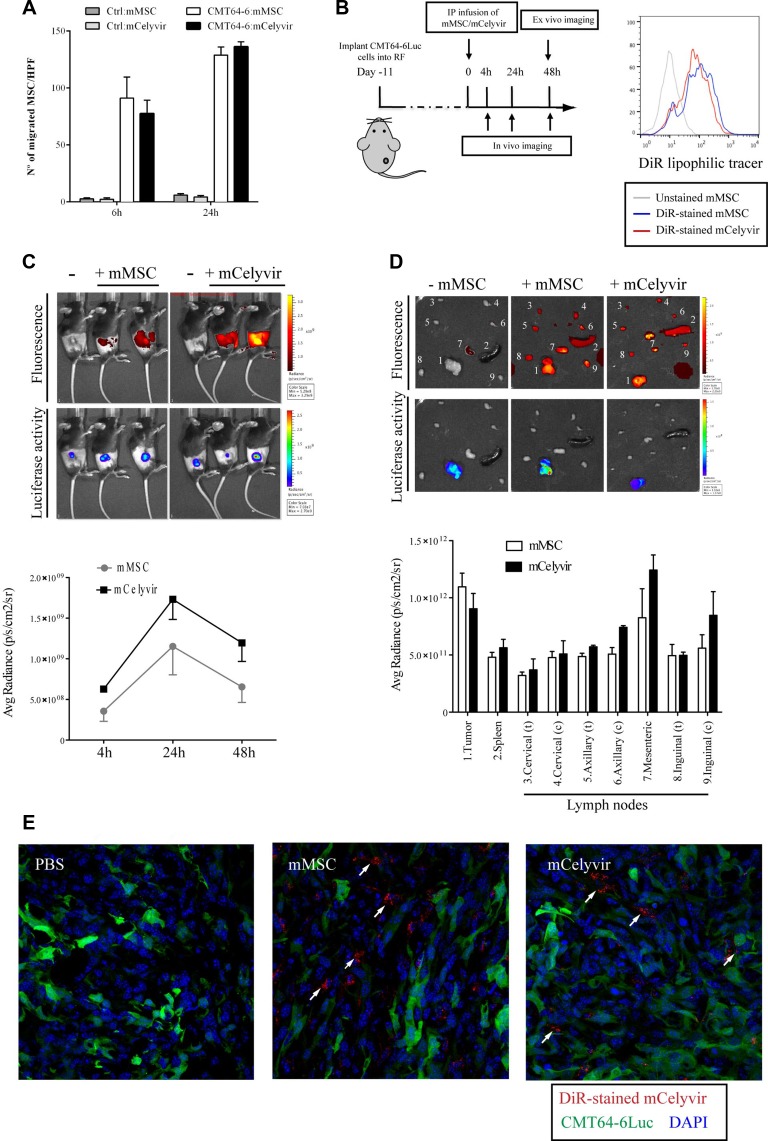
Homing of mMSC/mCelyvir to CMT64-6 tumors (**A**) *In vitro* cell migration assays of mMSCs/mCelyvir against CMT64-6 cells were performed using transwells with 8 μm pore filters. mMSCs/mCelyvir were transferred to the upper chambers. Cells were incubated in the presence of CMT64-6 cells in the bottom chambers, or DMEM (as negative control), for 6 h or 24 h. Migrated cells were fixed, stained with crystal violet and manually counted. The graph shows the average number of migrated mMSCs/mCelyvir cells in 10 HPF (high-power field). Bars represent mean of triplicates ± SEM. (**B**) *In vivo* experiment scheme (left): CMT64-6Luc cells were implanted in the right flanks of mice. After 11 days, mMSCs or mCelyvir previously stained with fluorescence tracker DiR were i.p. infused. The homing of mMSCs/mCelyvir to tumors was monitored at 4, 24 and 48 hours post-injection of mMSCs/mCelyvir to obtain serial fluorescence images. For visualizing CMT64-6Luc tumors, mice were intravenously injected with luciferin 3 minutes before imaging. In the time point 48 h, the mice were sacrificed and *ex vivo* imaging of tumors, spleen and lymph nodes was performed. Both, the fluorescent and bioluminescent imaging analysis were conducted with the IVIS 200 *in vivo* imaging system. In the right panel it is shown the analysis by FACS of the staining of mMCSc and mCelyvir with DiR. X-axes represent fluorescence intensity; grey line is from non-stained mMSC. (**C**) The upper panel shows *in vivo* fluorescence images of DiR labeled-mMSCs or DiR labeled-mCelyvir and the lower panel bioluminescence luciferase activity images of CMT64-6Luc cells 24 h post-mMSC infusion. As negative control for fluorescence we used mice non-infused with mMSCs. The images are from a representative experiment. In the graph it is represented the average radiant efficiency of fluorescence values, which indicates that DiR-mMSCs accumulated in the tumor tissues and reached peak value at 24 h post-injection. Experiment was repeated 3 times (*n* = 6 mice per group each time) and the graph is representative of one of them. (**D**) *Ex vivo* fluorescence images of dissected organs and tumors in the control mouse and DiR-mMSC- or DiR-mCelyvir-injected mice at 48 h post-injection (upper panel). Bioluminescence images are shown in the lower panel. Numbers in the tumors/organs coincide with those indicated in the graph, which shows the average radiant efficiency of the fluorescence in the tissues of the test groups and control group. (**E**) Tumor samples were analyzed using a confocal microscope, arrows point out DiR^+^ cells.

In order to validate CMT64-6 cells as a tumor model to analyze mCelyvir efficacy, we next checked whether these stem cells are able to home to CMT64-6 cells. We first performed *in vitro* transwell migration assays to evaluate the migration ability of mMSC. Interestingly, migration of ICOVIR5-infected mMSC cells (mCelyvir) to CMT64-6 cells was similar to that shown by uninfected mMSCs at 6 and 24 hours (Figure 3A). We also analyzed if mMSCs/mCelyvir migrate regardless of the cell type present in the bottom chamber. mMSCs/mCelyvir migrate towards mouse tumor cells (CMT64-6, RENCA and B16 cells) and primary fibroblast (MEFs), but the number of migrated mMSCs/mCelyvir depends specifically on target cell type. (Supplementary Figure 3). Our *in vitro* data indicate that mMSC/mCelyvir actively migrate toward CMT64-6 cells.

To assess the *in vivo* migration capacity of mMSCs/mCelyvir towards CMT64-6 tumors, we established subcutaneous tumors of CMT64-6 cells lentivirally-transduced with F-luciferase (CMT64-6Luc/GFP cells) in C57BL/6 mice. Eleven days after tumor cell inoculation, mMSCs/mCelyvir were stained with the fluorescent dye DiR and intraperitonally (i.p.) injected into these mice. A portion of mMSCs and mCelyvir were analyzed by flow cytometry to confirm equivalent DiR staining before injection (Figure 3B). The *in vivo* homing of mMSCs/mCelyvir to tumors was monitored at 4, 24, and 48 h post-injection to obtain serial fluorescence images using the IVIS system. The highest level of homing was consistently found in tumors at 24 hours for both mMSCs and mCelyvir (Figure 3C). There were no significant differences between mMSCs and mCelyvir homing to tumors. Interestingly, DiR positive cells were also detected in other organs related with immune activation, such as spleen and lymph nodes, although the presence in those organs was generally at lower levels than in tumors (Figure 3D). The presence of DiR-stained mMSC or mCelyvir inside tumors was confirmed by confocal microscopy (Figure 3E).

### *In vivo* mCelyvir inhibit the growth of CMT64-6 tumors

**Figure 4:**
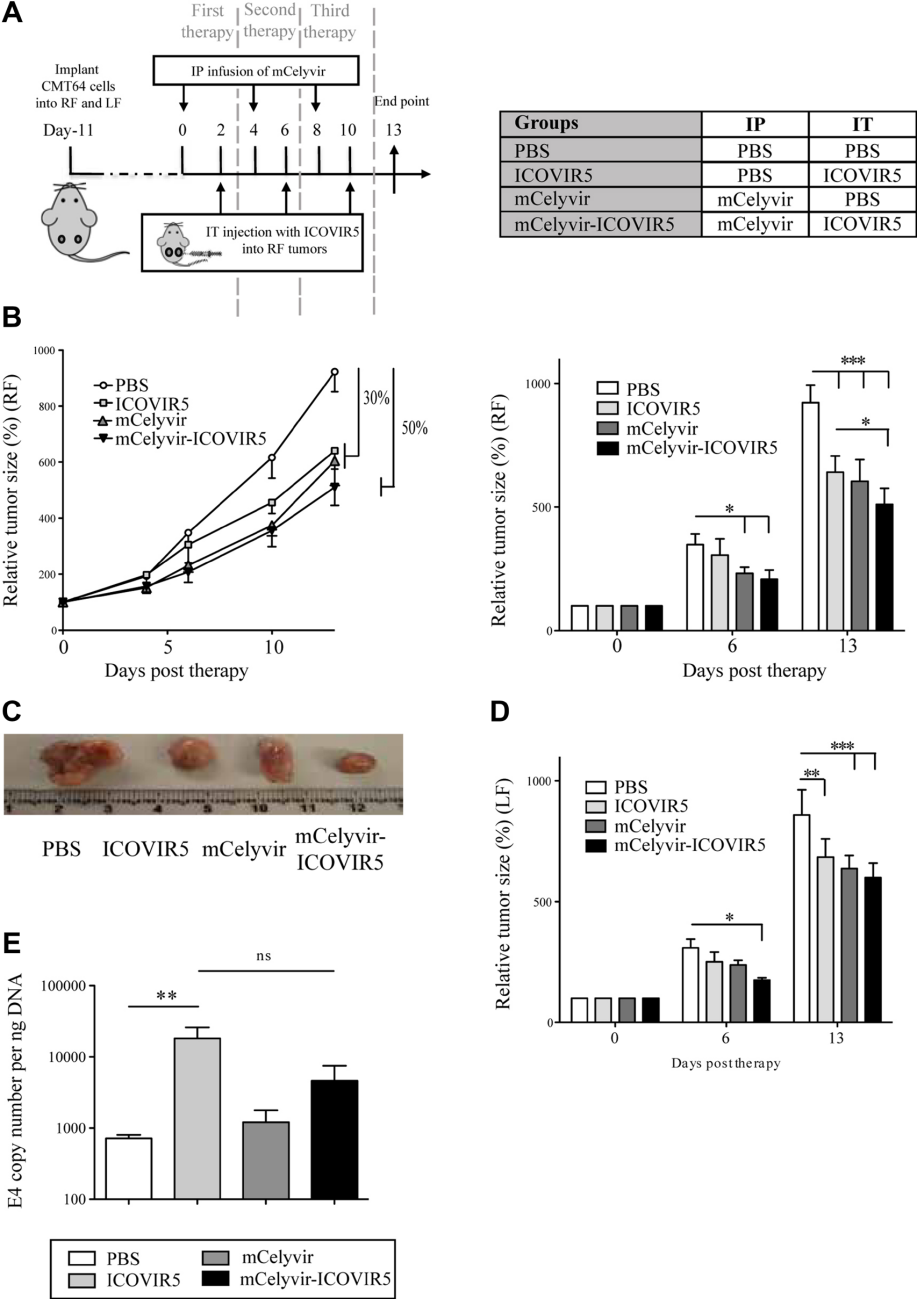
Anti-tumor activity of mCelyvir in the syngeneic mouse model of CMT64-6 tumors (**A**) *In vivo* experiment scheme: CMT64-6 cells were inoculated subcutaneously in C57BL/6 mice. Animals were treated i.p. with mCelyvir/PBS, and 48 h later with ICOVIR5/PBS (i.t.). Subsequently, the animals were investigated for antitumoral responses by measuring the tumoral volume. (**B**) For *in vivo* tumor volume experiments, tumors were measured periodically with a caliper, and volume was calculated. Both graphs show relative size of right flank tumors. (**C**) Representative photographs of tumors reflecting size at the end point (13 days). (**D**) Graph shows relative size of left flank tumors, as a readout of therapeutic distant effect. (**E**) Right flank tumors were harvested from each animal and the amount of viral DNA was measured by quantitative PCR (E4 copies). ANOVA was performed and statistical significance was defined as *P < 0.05, **P < 0.01 and ***P < 0.001.

To assay the therapeutic effect of mCelyvir, we established subcutaneous tumors of CMT64-6 cells in both right and left flanks of syngeneic C57BL/6 mice. We compared groups of mice treated i.p. with PBS or mCelyvir and, given the fact that mMSCs do not support the replication of human Ad, another group of mice were treated with Celyvir i.p. and two days later tumors were injected with saline or ICOVIR5, in order to mimic the dissemination of Ad into the tumors after infected mMSC inoculation [27]. Only tumors of the right flank were injected so the systemic therapeutic effect in the contralateral non-injected left tumor could be evaluated (Figure 4A).

Tumors were measured every three days and volume was calculated. We observed a similar tumor growth inhibition in ICOVIR5 and mCelyvir groups (30%) compared to PBS group, and a higher inhibition in the mCelyvir-ICOVIR5 group (50%) (Figure 4B, 4C). Interestingly, the tumors inoculated in left flanks, (non-injected i.t. with ICOVIR5), also showed a size reduction in the three treatment groups when compared to the control group; with a slightly higher tumor growth inhibition in mCelyvir-treated groups (Figure 4D). However, tumors were still growing over time until the end of experiments.

In order to confirm the ICOVIR5 replication in injected tumors, we analyzed the copy of Ad genomes by qPCR. Only tumors of groups treated with the injected ICOVIR5 showed a moderate amplification of Ad E4 gene and there were no differences between ICOVIR5 and mCelyvirICOVIR5 treated mice (Figure 4E). Ad genomes were not detected in left flank tumors (data not shown), indicating that the volume reduction was not a direct effect of virusmediated oncolysis.

We could observe that the treatment of tumor-bearing animals only with i.p.-injected mCelyvir inhibited tumor growth despite the lack of ICOVIR5 replication in mMSC. Because it is well known that MSCs *per se* could positively or negatively modify the tumor progression, we compared the effect of mCelyvir and non-infected mMSCs in this CMT64-6 tumor model. Our results showed that mMSCs had no effect over tumor growth, while mCelyvir again induced a significant reduction in tumor size (Supplementary Figure 4), indicating that their antitumor effect was due to the combination of Ad-infected mMSCs and Ad, and not to mMSCs alone.

Overall these findings demonstrate that both ICOVIR5 and ICOVIR5-infected mMSCs provide therapeutic benefit in the treatment of CMT64-6 tumors, and that the combination of both exhibits a higher therapeutic effect, suggesting that mMSCs carrying ICOVIR5 convey more treatment benefit than just serving as a viral carrier alone.

### *In vitro* mCelyvir do not affect CMT64-6 viability, but produce pro-inflammatory cytokines

**Figure 5:**
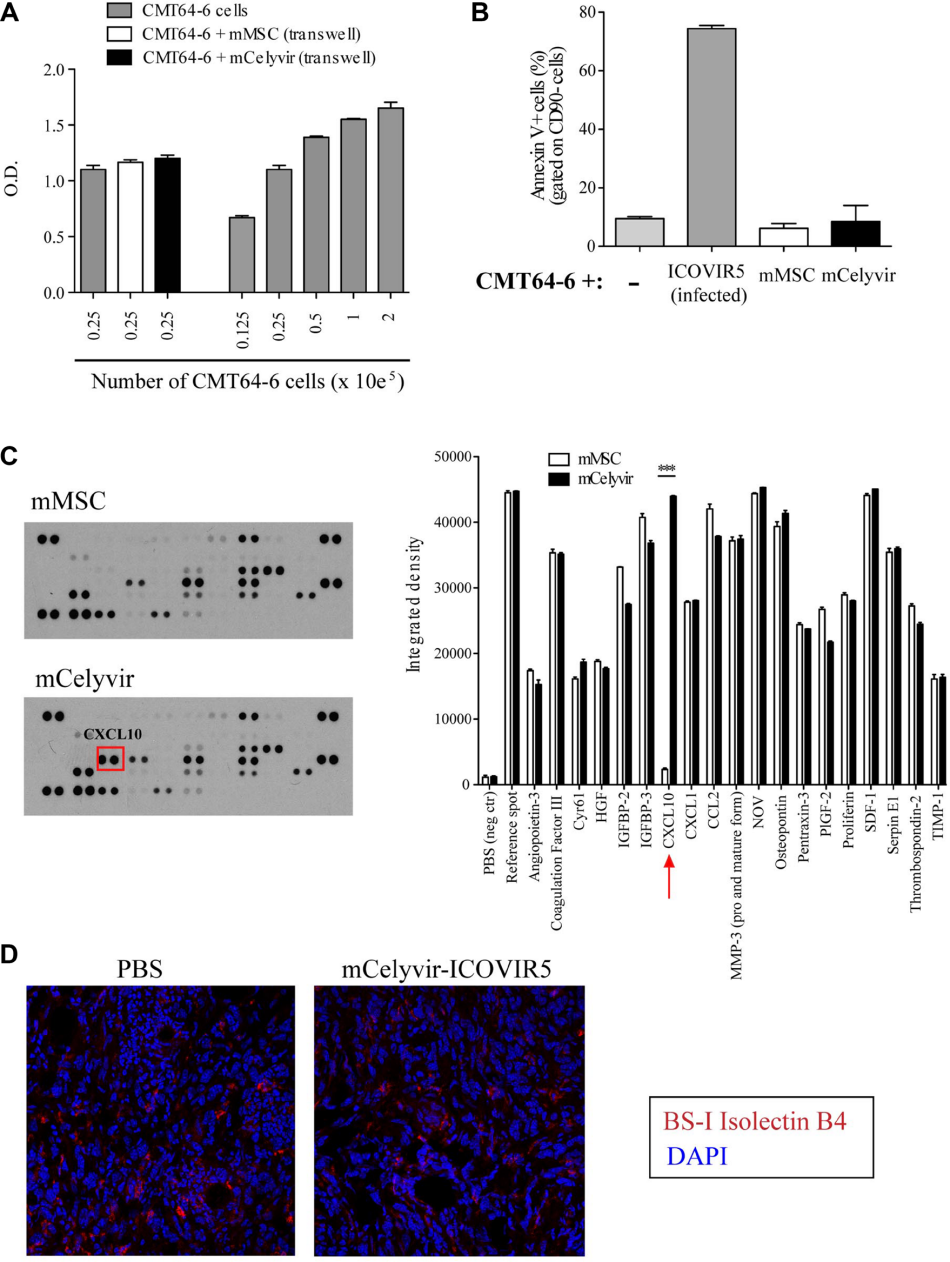
Study of mechanisms of action of Celyvir on CMT64-6 (**A**) CMT64-6 cells were cultured in the presence of factors produced by mMSC/mCelyvir in transwell with of 0.4 μm pore size. CMT64-6 cell number was measured by MTT after 3 days. A serial dilution curve of CMT64-6 cells was used as a standard curve. (**B**) CMT64-6 cells and mMSCs/mCelyvir were cocultured during 3 days and the cell death was measured by the proportion of apoptotic cells (Annexin V^+^ cells) within the CD90^−^ subset by flow cytometry. ICOVIR5 Infected CMT64 cells were also analyzed (as a positive control). (**C**) Supernatants from mMSC/mCelyvir after 24 h of culture were analyzed for the presence of angiogenesis-related proteins. The arrays show the pattern of each condition. The graph shows pixel intensity quantification of the dots corresponding to each molecule measured. (**D**) Sections from tumors of mice being treated with PBS or with mCelyvir-ICOVIR5 were stained for BS-I Isolectin B4 by immunofluorescence for blood vessel density evaluation (red). Student's *t* test was performed and statistical significance was defined as ***P < 0.001.

To further investigate the therapeutic effect of mCelyvir in CMT64-6 tumor treatment, we studied a possible direct toxicity of mCelyvir to CMT64-6 cell line by performing coculture experiments. First, we performed transwell assays using microporous inserts of 0.4 μm pore size that allow the passage of small molecules but not cells. mMSCs or mCelyvir were plated in the upper compartment, while CMT64-6 cells were plated in the lower compartment, to assay the effect of molecules secreted by mMSCs/mCelyvir in CMT64-6 cell viability. After 3 days, we evaluated the number of CMT64-6 cells using a MTT assay. No differences were observed in cocultures compared to CMT646 cultures (Figure 5A).

In order to determine whether cell-cell contact is necessary for a possible toxic effect, mCelyvir or mMSC were cocultured with CMT64-6 for 3 days. Annexin V staining was analyzed by flow cytometry. No significant differences were observed in the percentage of apoptotic CMT64-6 cells (CD90- Annexin V+) when cocultured with mMSCs or mCelyvir. By contrast CMT64-6 died by apoptosis when infected with ICOVIR5 (Figure 5B). These experiments indicate that the *in vitro* coculture of CMT64-6 with mCelyvir does not affect the growth or viability of the tumor cells. We next examined if the therapeutic effect of mCelyvir could be due to anti-angiogenesis effects. To analyze the possible anti-angiogenic role of mCelyvir, we analyzed the expression profile of angiogenesis-related proteins by mMSCs in their basal state and after ICOVIR5 infection (mCelyvir) (Supplementary Figure 5A and 5C). Most noticeable was the expression pattern of the antiangiogenic chemokine CXCL10 (IP-10), which was not expressed by intact mMSCs but it was produced by ICOVIR5-infected mMSCs (Figure 5C). Moreover, we studied tumor angiogenesis by immunohistochemistry. Staining of endothelial cells with Isolectin B4 (BSI-B4) revealed no changes in the number or morphological distribution of vessels in the tumors (Figure 5D).

**Figure 6:**
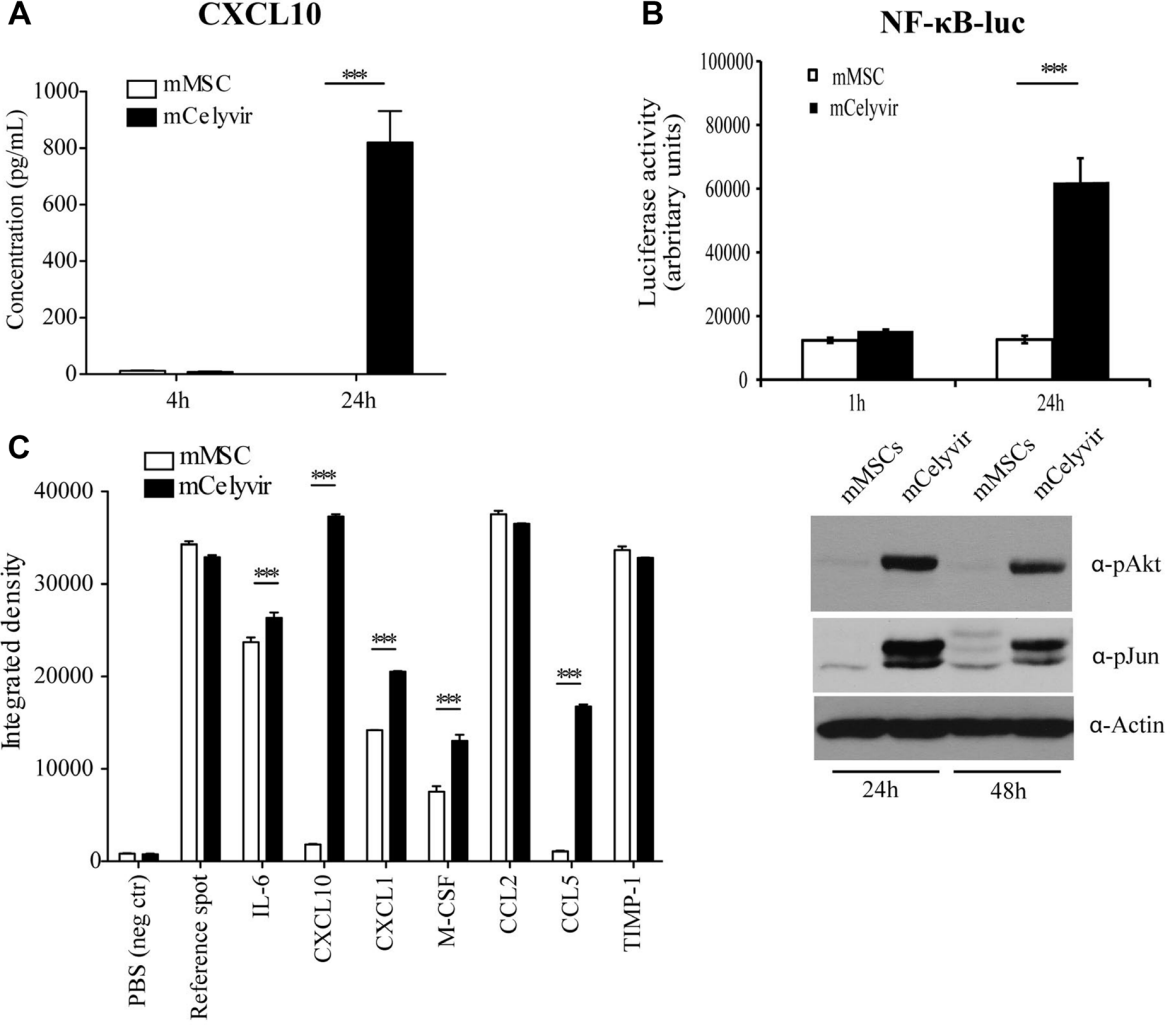
Cytokines secreted by mMSC vs mCelyvir (**A**) Quantification of CXCL10 production on mMSC/mCelyvir after 24 h by ELISA CXCL10. (**B**) Up panel, mMSC were transduced with a lentiviral system for detecting differences in activation of NF-kB levels by luciferase expression after ICOVIR infection (1 h/24 h). The graph shows luciferase activity. Down panel, Western Blott performed with phospho-Akt and phospho-Jun specific antibodies on mMSC and mCelyvir after 24 h or 48 h of Ad infection. Anti-actin antibody was used as a loading control. (**C**) Pixel intensity quantification of the array blot (original shown on Supplementary Figure 5) for the evaluation of the production of different key factors of the immune response. Student's *t* test was performed and statistical significance was defined as ***P < 0.001.

The upregulation of CXCL10 secretion after Ad infection of mMSCs was confirmed using an ELISA assay, which revealed levels of 800 pg of CXCL10 per mL of supernatant, 24 h after infection (Figure 6A). Previous studies have shown that Ad infection induces the expression of CXCL10 in mouse kidney epithelium-derived cells in a NF-kB-dependent manner. The mechanism underlying the upregulation of CXCL10 is dependent on Akt signaling [28]. To analyze whether NF-kB is activated in mMSCs after ICOVIR5 infection, we transduced these cells with a NF-kB promoter driven luciferase reporter system for detecting NF-kB activation [29]. When infected with ICOVIR5 (mCelyvir), cells exhibited a dramatic increase in luciferase activity, indicating NF-kB-driven luciferase expression (Figure 6B). We also confirmed the activation of Akt by Western Blot, using phospho-specific Akt antibody. In addition, we found an increase in the activation of phospho-Jun after ICOVIR5 infection (Figure 6B).

NF-κB is a transcription factor that plays a key role in modulating the immune response to infection, regulating innate and adaptive immune response genes. To determine whether infection of mMSCs with ICOVIR5 could trigger an immune system response, we compared the expression profile of a panel of factors involved in immune activation from mMSCs or mCelyvir (Figure 6C and Supplementary Figure 5B). Again, an increase of CXCL10 expression after Ad infection was observed. Furthermore, a higher expression of other proteins with inflammatory and chemoattractant activity, such as CCL5 (RANTES), CXCL1, M-CSF, and IL-6 (Figure 6C) was also detected. Interestingly, most of those cytokines have been previously shown to be regulated by NF-kB [30].

### mCelyvir induce immune infiltration in CMT64-6 tumors

**Figure 7:**
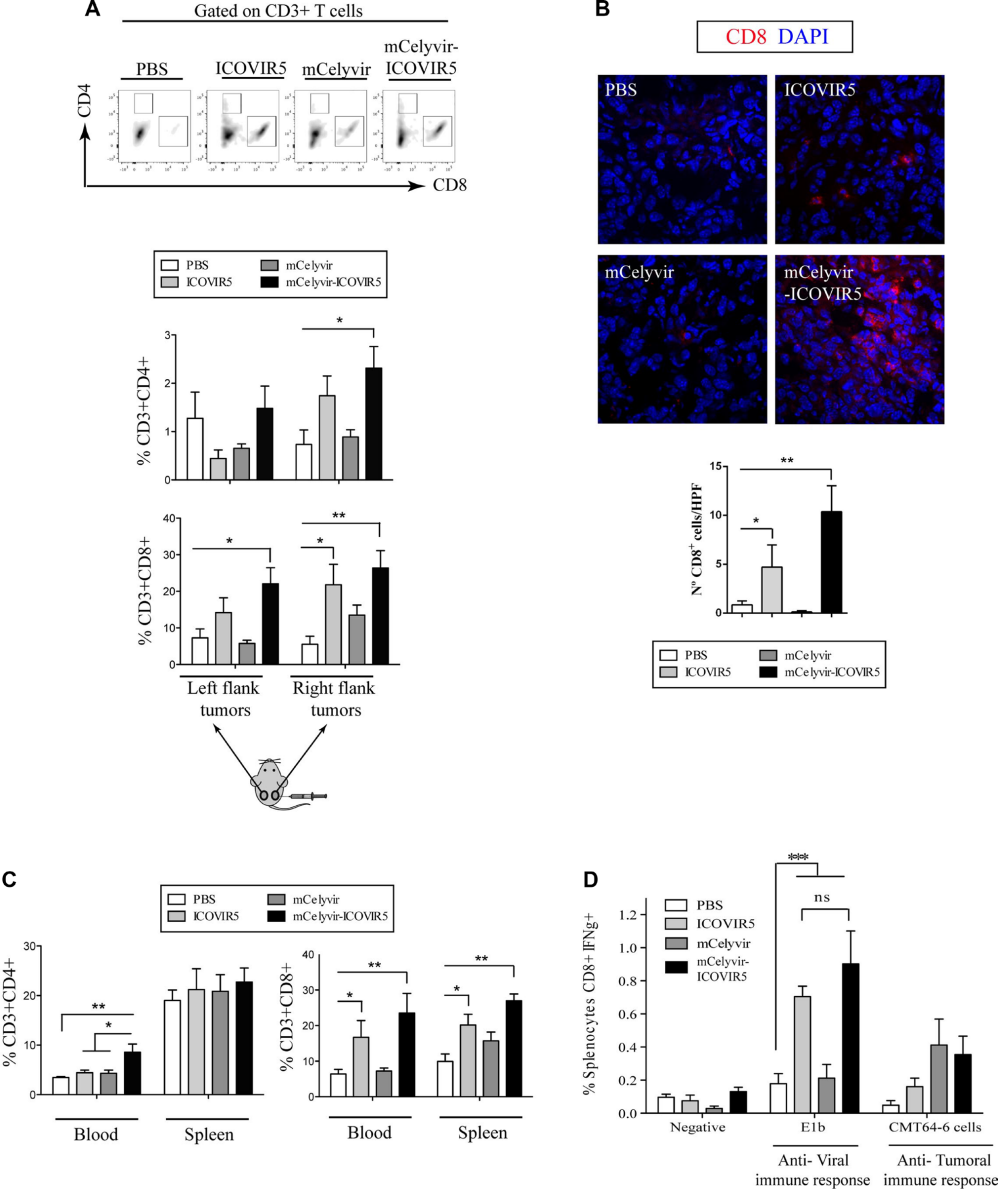
mCelyvir-ICOVIR5 treatment increases tumor lymphocyte infiltration (**A**) Right and left flank tumors of mice treated as explained in Figure 4 were disaggregated and percentages of CD8^+^ and CD4^+^ T cells (within CD3^+^ cell subset) were analyzed by FACS. (**B**) Immunohistochemistry of tumors confirmed higher number of CD8^+^ lymphocytes infiltration (red) in ICOVIR5- and mCelyvir-ICOVIR5- treated groups. DAPI counterstaining (blue). (**C**) Percentages of CD8^+^ and CD4^+^ T cells (within CD3^+^ cell subset) were analyzed by FACS on blood and spleen of mice of each group. (**D**) Proportion of CD8^+^ IFNγ ^+^ splenocytes upon different stimulations (a major adenoviral peptide E1b or a lysate of CMT64) was determined by FACS in the splenic CD8^+^ cells of mice of each group. This experiment was performed three times and a representative one is shown. Results were analyzed using one way ANOVA followed by Tukey's post hoc test. Statistical significance was defined as *P < 0.05, **P < 0.01 and ***P < 0.001.

Our previous results show a notable increase in CXCL10 secretion, among other proinflammatory cytokines, in mMSCs when infected with ICOVIR5. CXCL10 is a chemoattractant for activated T cells. *In vitro* CXCL10 induces the migration of mouse lymphocytes (Supplementary Figure 6A) and *in vivo* preferentially attracts activated lymphocytes to the areas of inflammation and tumors [31]. To further investigate a proinflammatory role of mCelyvir *in vivo*, we evaluated whether the mCelyvir treatment of CMT64-6 tumors would lead to an increase lymphocyte infiltration in treated tumors. Flow cytometry analysis of tumors revealed that percentages of tumor-infiltrating CD8^+^ and CD4^+^ T cells were higher in right flank tumors of mice treated with the combined treatment (mCelyvir-ICOVIR5) (Figure 7A and Supplementary Figure 7A). In the groups of tumors treated with ICOVIR5 (i.t.) a significant increase of CD8^+^ T cells was detected in the right flank tumors (Figure 7A). These results were corroborated by CD8^+^ T cell-immunohistochemistry analysis (Figure 7B). Interestingly, we also found a higher presence of CD45^+^ cells in the groups of mice treated with ICOVIR5 and /or mCelyvir (Supplementary Figure 6B). Moreover, in the non-treated left flank tumors a significant increase in CD8^+^ T cells infiltration was detected only in mCelyvir-ICOVIR5 group (Figure 7A).

We also analyzed the lymphocyte subpopulations in peripheral blood and spleen. In mice treated with mCelyvir-ICOVIR5 an increase of CD8^+^ and CD4^+^ lymphocytes were detected in peripheral blood and only of CD8^+^ in spleen (Figure 7C). In mice treated only with ICOVIR5, a moderate increase in CD8^+^ cells was also detected in peripheral blood and spleen, but no changes were found in CD4^+^ lymphocytes (Figure 7C).

Since CD8^+^ cells were the higher infiltrating lymphocyte population, especially in the group of mice treated with mCelyvir-ICOVIR5, we decided to study the *ex vivo* IFNγ production upon different stimulations in spleen-derived CD8^+^ T cells obtained from treated mice. To test the induction of anti-Ad immunity, IFNγ secretion was analyzed upon stimulation with a peptide from E1b (VNIRNCCYI) (Supplementary Figure 7B and 7D). Only animals of groups treated with ICOVIR5 (i.t.) showed T lymphocyte activation against E1b (Figure 7D). However, there was no difference in anti-Ad CD8^+^ cell activation in the group treated only with ICOVIR5 compared to the group treated with the combined therapy mCelyvir-ICOVIR5. Interestingly, the proportion of splenocytes CD8^+^IFNγ^+^ activated against CMT64-6 tended to increase in both mCelyvir-treated groups (Figure 7D).

Altogether these results demonstrate a correlation between the lymphocyte increase in tumors, spleen and blood and the therapeutic effect of ICOVIR5 when administered alone or with mCelyvir, suggesting that the administration of both therapeutic agents triggers a higher antitumor immune response.

## DISCUSSION

Clinical trials with oncolytic Ad point to a limited efficacy due to insufficient viral delivery to tumor and to host-antiviral immune responses [3]. The use of MSCs as vehicles for oncolytic Ad has been shown to increase its efficacy [8]. Several groups, including ours, have explored the possibility of using those cells in animal models as vehicles to avoid aforementioned limitations. Currently two trials are ongoing using MSCs as cell carriers for oncolytic virotherapies, one using an oncolytic measles virus (NCT02068794), and our clinical study where we use ICOVIR5 (NCT01844661). We previously performed an exploratory study in children with refractory metastatic neuroblastoma based on the intravenous infusion of human Celyvir. The tolerance to the treatment was excellent and clinical responses were documented including complete remissions [12, 16].

Based on our experience using MSCs as antitumor agents, we hypothesized that MSCs behave more than just simple cell carriers, e.g. inducing antitumor immune activation. Adenoviral infection induces the expression and release, *in vivo* and *in vitro*, of proinflammatory cytokines, including IL-6, IL-8, tumor necrosis factor alpha (TNF-α), macrophage inflammatory protein-1 alpha (MIP-1α), CCL5 (RANTES) and interferon-gamma (IFNγ) [32]. Using murine MSCs and a human Ad, as ICOVIR5, we did not observe any expression of TNF-α, MIP-1α and IFNγ; but we found overexpression of CXCL10, CCL5, IL-6 and M-CSF. Transcriptional regulation of CXCL10 following infection with Ad vectors, differs significantly from the transcriptional mechanisms activated in response to TNF-α and IFNγ [33]. In addition, in kidney-derived epithelial cells, RGD-modified Ad (as ICOVIR5) rapidly activate Akt that contribute to the Ad induction of CXCL10 through its effects on NF-kB pathway [28]. Ad activation of MAPK and ERK signal transduction pathways, in a post-internalization step of viral entry, contributes to CXCL10 expression. Some of these mechanisms could be occurring in mMSCs after Ad infection, inducing the CXCL10 and CCL5. Strikingly, the production of these chemokines by MSCs have been strongly related with the polarization of these cells to proinflammatory and antitumoral phenotype [34, 35].

mCelyvir home to the tumor during the first 24 hours. However, the production of inflammatory proteins after Ad infection of mMSCs is unlikely to induce a high number of tumor infiltrating lymphocytes (TILs). Our data of immune cells infiltrating the tumors indicate that mCelyvir alone is not sufficient to increase the number of TILs, but when we combined mCelyvir with ICOVIR5 (i.t.) a higher increase of TILs was detected than in mice treated only with ICOVIR5. These data suggest that some level of intra-tumor Ad-replication could be necessary for an effective TILs presence, a hypothesis supported by other immunocompetent mouse models [36]. Moreover, in our model, the higher number of TILs correlates with a better antitumor effect in the group of mice treated with combined therapy mCelyvir-ICOVIR5, suggesting a main role of antitumor immune activation for the efficacy of this therapeutic strategy.

A major limitation in the advancement of this field is the lack of adequate immune- and Ad replication permissive-competent models. Our group previously studied a cotton rat animal model, that is a semi-permissive host for Ad replication [10]. We used rat MSCs (rMSCs) loaded with human oncolytic Ad to evaluate the anti-adenoviral immune response in mice treated with oncolytic Ad or with oncolytic Ad-infected rMSCs. Ad-loaded rMSCs reduced *in vivo* the amount of anti-Ad antibodies and *ex vivo* the splenocyte IFNγ production. We also studied the effect of Ad-loaded rMSCs in subcutaneous tumors generated in the flank of the cotton rats by injecting LCRT fibrosarcoma cells. We observed that tumors injected with Ad-loaded rMSCs, exhibited higher amount of viral DNA than tumors injected only with naked Ad [10]. However, in that study, we couldn't find a correlation between antitumor efficacy and treatment with oncolytic Ad or Ad-loaded rMSCs. In the present study there were no differences in the amount of Ad genomes or anti-Ad CD8^+^IFNγ^+^ splenocytes between ICOVIR5 and mCelyvir-ICOVIR5 groups. In this sense, it should be noted that rMSCs, but not mMSCs, are permissive to human Ad infection and replication [10, 37]. Moreover, Ad-loaded rMSCs maintained their immunosuppressive properties. In contrast, human Ad infection of mMSCs induced apoptosis. Interestingly, when we tested T lymphocyte activation against CMT64-6 extracts, a slight increase was detected in both mCelyvir-treated groups. We assessed the tumor-specific immune responses using CMT64-6 cells transduced with GFP-expressing lentiviral vectors, and then tested T lymphocyte activation against GFP peptides as a model of tumor-like antigen; however, we did not detect T cell activation (data not shown). Although we have not demonstrated an increase in the antitumoral activation of T cells in mice treated with mCelyvir, we cannot exclude that this is actually happening in our model.

The higher antitumor effect of the combined mCelyvir-ICOVIR5 therapy in our model is very interesting because mMSCs do not produce new adenoviral particles after Ad infection (in fact they undergo apoptosis), but are still triggering an antitumor response. A potential mechanism of action would be that adenoviral particles are transmitted to tumor cells without an extracellular transmission. Hamada *et al*. (2007) demonstrated that A549 cells bearing replication-competent Ad delivered Ad through cell fragments, originated by apoptotic blebbing, to cancer cells [38]. They observed engulfment of cell fragments only in proliferative malignant cells and not in non-proliferative normal cells. In addition, their carrier cell-mediated adenoviral infection produced cytotoxic T lymphocyte responses to tumoral cells as well as Ad [38]. Here, we did not observe a clear CD8^+^ cell infiltration in tumors of mice treated only with mCelyvir, but we observed a minor increase of CD8^+^IFNγ^+^ in splenocytes of this group.

In summary, our results not only demonstrate the capacity of mCelyvir to migrate toward tumors but also document a new role for MSCs when used as cell carriers of oncolytic Ad based on their ability to produce proinflammatory cytokines. These data show how the use of MSCs as carriers of oncolytic Ad can be beneficial for two reasons: first, it is a way to directly transport the Ad to the tumors and second, the infected MSCs can increase the antitumor response working collaboratively with the action of the oncolytic Ad. These findings have implications for patients with metastatic cancer treated with oncolytic virotherapy.

## MATERIALS AND METHODS

### Cell culture

Cells were cultured in Dulbecco's Modified Eagle's Media supplemented with 10% fetal bovine serum (FBS) as well as streptomycin (100 mg/mL) and penicillin (100 U/mL) at 37°C in a humidified atmosphere with 5% CO_2_. The murine non-small-cell lung carcinoma cell line CMT64 was kindly gifted by Dr. Sthepan Kubicka from Hannover Medical School (Hannover, Germany). The clone CMT64-6 was isolated from the cell line CMT64. The clones were obtained by diluting 5 cells in 1ml and seeding 200 ml per well. We amplified the cells and we infected monolayers (4 × 10^5^) of the different clones with ICOVIR15 with 200 MOI. Five days post-infection we obtained a cellular extract and quantified the virus by hexon protein staining [39]. We also infected parental cell line and clone 6 with different MOIs of AdTL and evaluated transduction on a fluorescence microscopy. CMT64-6 cells were transduced with a replication-deficient lentiviral vector containing firefly-luciferase and GFP expression cassettes (provided by Dr A. Rodríguez, Department of Molecular Biology, Universidad Autónoma de Madrid, Spain) for *in vivo* homing experiments (referred to as CMT64-6Luc/GFP). HEK293, B16 and RENCA cells were obtained from the American Type Culture Collection (ATCC, Manassas, VA). Human MSCs were obtained from Lonza (Basel, Switzerland). mMSCs were obtained as previously described [26]. Briefly, abdominal adipose tissue from C57BL/6 mice was digested with collagenase IV (Sigma-Aldrich, Madrid, Spain), filtered through a sterile 70μM nylon mesh cell strainer (Fisher Scientific, Waltham, MA) and cultured in MesenCult medium (Stem Cell Technologies, Vancouver, Canada). Non-adherent cells were discarded through subsequent culture passages. The mMSCs characterization was verified by: (I) fibroblast morphology of adherent cells; (II) flow cytometry expression profile analysis using monoclonal antibodies to mouse CD44 (clone IM7), CD90 (clone 52-3.1), Sca1 (clone D7), CD11b (clone M1/70), CD45 (clone 30-F11) (eBioscience, Barcelona, Spain), and CD11b (BD Bioscience, Madrid, Spain); and (III) verification of multilineage differentiation potential in adipogenic, osteogenic and chondrogenic phenotype by culture mMSCs in specific cell culture media (Lonza, Barcelona, Spain) followed by lipidic drop (oil red O), bone ECM (alizarin red) and chondrogenic glycosaminoglycans in the presence of TGF-β (alcian blue) staining respectively (Supplementary Figure 1). mCelyvir were mMCs infected with ICOVIR5 at an MOI of 200 p.f.u. per cell.

### Oncolytic adenoviruses

ICOVIR5 has been extensively described elsewhere [22, 23, 40]. Briefly, ICOVIR5 (Ad-DM-E2F-K-Delta24RGD) is an optimized oncolytic Ad that combines E1a transcriptional control by an insulated form of the E2F promoter with the Delta24 mutation of E1a to improve the therapeutic index of AdDelta24RGD. ICOVIR5 also contains the Kozak sequence at the E1a start codon, which is important to restore E1a expression and viral replication to AdwtRGD levels in tumor cells. AdTL vector, containing recombinant fiber-RGD protein and expressing the firefly luciferase and EGFP, was described previously [24, 25]. It was used to measure transduction efficiency by FACS or fluorescence microscopy. ICOVIR15Luc was constructed by Dr. Luis Rojas (Catalan Institute of Oncology-IDIBELL) and it will be described elsewhere. This virus contains a splicing acceptor signal connected to the firefly luciferase gene inserted after the fiber gene of ICOVIR15 [41]. To quantify the viral replication, cells were infected with different MOIs of ICOVIR5. Cells were collected 72 hr after infection and the total DNA was isolated using a DNeasy tissue kit (Qiagen, Valencia, CA) and quantified using a spectrophotometer (NanoDrop; Thermo Scientific, Pittsburgh, PA). Adenoviral E4A gene expression was quantified via quantitative PCR (qPCR) with iQ SYBR Green supermix (Bio-Rad, Hercules, CA), using primers and protocol described elsewhere [42, 43]. Each sample was analyzed in triplicates. Results are presented as E4A copy number/ng of DNA. In ICOVIR15Luc-infected cells, luciferase activity was measured in the lysates of infected cells using Luciferase Assay System (Promega, Madrid, Spain). Luciferase expression was also analyzed in the lysates by western blotting, that was conducted using standard protocols as described previously [44]. Primary antibodies employed were monoclonal anti-luciferase antibody and mouse monoclonal anti-tubulin antibody (clone LUC-1 and clone DM1A respectively, Sigma-Aldrich). To quantify infectious viral progeny of CMT64-6 cells, mMSCs, hMSC, HEK-293 (as permissive positive control) and B16 (as non-permissive negative control) supernatants of infected cells were collected 96 h after infection and incubated with HEK293 cells in serial ten-fold dilutions, as per the Adeno-X Rapid Titer Kit protocol (Clontech, Mountain View, CA). After 48 h, the cells were fixed/permeabilized with methanol and stained for hexon plaques and viral titer was determined according to the manufacturer's protocol. The titration units (infection unit/mL) values quantified through this protocol are similar to plaque forming units (pfu). To analyze the toxicity effect of ICOVIR5 on mMSCs, cell viability was evaluated by trypan blue exclusion method at 24 h, 48 h and 72 h *post*-infection (200 MOI). There was a total of 3 wells per condition. The experiment was repeated 3 times. Apoptosis at 48 h *post*-infection time point was measured using Annexin V detection kit (BD Bioscience) and then analyzed by flow cytometry (FACSCalibur; Becton-Dickinson, Mountain View, CA, USA).

### mMSCs-CMT64-6 cell coculture and cytotoxicity assay

To evaluate *in vitro* the toxicity of mCelyvir to CMT64-6 cell line, mMSCs were infected with ICOVIR5, and, after 2 h, Ad was removed by washing out twice with PBS. CMT64-6 cells were added to the wells and cocultured during 3 days (or 5 days, data not shown). Cells were then stained with CD90 (clone 5E10, eBioscience), and Annexin V and apoptosis in CD90-negative cells (CMT64-6 cells) were analyzed by flow cytometry (FACSCalibur). For transwell cultures (Figure 5A), 2.5 × 10^5^ mMSCS or mCelyvir were plated in the upper compartment (transwell 0.4 μm pore size; Corning, NY), while the same number of CMT64-6 cells were plated in the lower compartment. A standard curve with different number of CM64-6 cells plated in the lower compartment, but without mMSCs/mCelyvir in the upper compartment, was also done. After 3 days, upper compartment was removed and cell viability assay was performed in CMT64-6 cells using MTT Kit according to manufacturer's instructions (Roche, Basel, Switzerland). Each value from these indicated assays represents the mean ± SEM of triplicate measurements from two independent experiments.

### Luciferase assay

We determined the activation of NF-κB by using a luciferase reporter system [29]. Replication incompetent lentiviral vectors were created using the pHAGE NF-κB-TA-LUC-UBC-GFP-W plasmid, a gift from Darrell Kotton (Addgene plasmid # 49343). The plasmid encodes the NF-κB consensus binding sequence upstream of the minimal TA promoter of the herpes simplex virus followed by the firefly luciferase gene, as well as the GFP gene upstream of the ubiquitin-C promoter. Transduction of mMSCs was performed overnight and GFP expression was assessed by flow cytometry. For luciferase-reporter assays, cells were infected with ICOVIR5 and, 24 hours later, 10^4^ cells were lysed and luciferase activity was assayed with the luciferase assay system (Promega), according to the manufacturer's instructions. Activation of Akt and c-Jun was analyzed in the lysates of these cells by western blotting using the following mouse monoclonal antibodies: anti-phospho-Akt-Ser473 (clone 2118, Epitomics), anti-phospho-c-Jun (clone KM-1, Santa Cruz Biotechnology) and anti-Actin (clone AC-15, Sigma-Aldrich).

### Animal experiments

We established subcutaneous tumors of CMT64-6 or CMT64-6Luc cells injecting 5×10^6^ cells (or 2.5 × 10^6^ cells for *in vivo* imaging) into the flanks of 6–8 weeks old C57BL/6 syngeneic mice. Tumors were grown for 10–12 days to a size of 6–7 mm in diameter. The studies were approved by Spanish “Animal Ethics Committee” in compliance with European Union Directives. Animal studies performed at the University of Chicago were approved by “UC Institutional Animal Care and Use Committee”. For the efficacy experiments we injected intraperitoneally mMSCs/mCelyvir in CMT64–bearing mice (1 × 10^6^ cells/mouse). For *in vivo* optical imaging of mMSCs/mCelyvir homing to CMT64-6Luc tumors, mMSCs/mCelyvir were incubated with 320 mg/mL DiR buffer for 30 min at 37°C according to the protocol of XenoLight DiR (Caliper Lifesciences, Hopkinton, MA). Then DiR-labeled mMSCs/mCelyvir were washed twice with PBS buffer and intraperitoneally injected in CMT64-6Luc tumor-bearing mice (2 × 10^6^ cells/mouse). Some cells were analyzed by flow cytometry for DiR staining. For visualizing CMT64-6Luc tumors, mice were intravenously injected with 150 mg/kg body weight of firefly luciferin (Promega, Madison, WI), 3 minutes before imaging and anesthetized with isofluorane during the procedure. The homing of mMSCs/mCelyvir to tumors was monitored using IVIS system at 4, 24 and 48 hours post-injection of mMSCs/mCelyvir to obtain serial fluorescence images. Both, the fluorescent and bioluminescent imaging analysis were conducted with the IVIS 200 *in vivo* imaging system (Caliper). Bioluminescence images of tumors were taken with the exposure time of 30 seconds. For *ex vivo* imaging, tumors, spleen and lymph nodes were collected at 48 h post mMSCs injection. Bioluminescent and fluorescent images of each mouse and sample were analyzed using Living Image software (Xenogen, Alameda, CA). Regions of interest (ROI) were drawn over the signals, and average radiant efficiency was quantified in p/s/cm^2^/sr. The experiment was performed 3 times (*n* = 5 mice per group each time). For *in vivo* tumor volume experiments, tumors were measured periodically with a caliper, and volume was calculated as (length × width^2^)/2 [45]. The experiment was repeated 3 times (*n* = 5 mice per group). At different time points mice were sacrificed and tumors and spleens were harvested and processed for flow cytometry, histology and E4A copy gene analysis.

### Immunofluorescence

Tumor samples were fixed in 10% buffered formalin solution and embedded in optimal cutting temperature (OCT) (Tissue-Tek) medium. Cryosections (8–10 mm) were stained for immunofluorescence. Immunofluorescent stains were applied using BS-I Isolectin B4 Biotin Conjugate (Sigma-Aldrich), monoclonal mouse CD8 (clone 53-6.7, eBioscience) and CD45 (clone 30-F11, Biolegend). Briefly, tumor sections were dried at room temperature (RT), washed three times with cold PBS and blocked with 10% BSA for 30 minutes. Samples were incubated for 2 hours at RT with primary antibodies, washed and incubated for 1 hour at RT with the secondary antibody. Antibodies were used between 5–10 μg/ml. After washing with cold PBS three times, tissues were covered with Prolong Gold anti-fade reagent with DAPI (Invitrogen). Images of antibody-stained sections were acquired using the Sp5 Tandem Scanner confocal microscope running LAS AF software (Leica Microsystems, Barcelona, Spain).

### Flow cytometry analysis of immune cell populations

Single cell suspensions were made from collagenase IV-digested tumors or spleen by mashing cells through a sterile 70 μM nylon mesh cell strainer using the rubber end of a 3 mL syringe into ice-cold PBS. Red blood cells were removed by treatment with ACK Lysis Buffer (Lonza) for four minutes on ice. Peripheral blood was collected on EDTA-coated tubes and treated with RBC buffer (Biolegend, San Diego, CA) to lyse red blood cells. For detection of surface antigens, the cells were stained with primary antibodies for 30 minutes at 4°C in fluorescence-activated cell sorting (FACS) buffer (0.5% bovine serum albumin, 0.05% sodium azide) in PBS. After the cells were washed, secondary antibodies were added in FACS buffer for another 30 minutes at 4°C. After fluorescent labeling, the samples were washed, acquired with the LSR II flow cytometer (BD Biosciences, Franklin Lakes, NJ) and analyzed using FlowJo analysis software (TreeStar, Cupertino, CA). The primary antibodies used were: monoclonal antibodies to mouse CD45 (clone 30F11), mouse CD3 (clone 145-2C11), CD4 (clone GK1.5), CD8 (clone 53-6.7), CD11b (clone M1/70) and CD11c (clone N418) or an appropriate negative control (isotype immunoglobulins) (eBioscience). All antibodies were used between 5–10 μg/ml.

### IFNγ-Intracellular staining

IFNγ production was analyzed by intracellular cytokine staining (ICS) to monitor CD8^+^ cells immune responses from splenocytes isolated from treated mice. Spleens were treated as above-mentioned and the splenocyte cell suspension was resuspended in RPMI medium (Gibco). One million of cells were seeded in wells of 96 well plate with U-bottom, where different stimulants were added: (i) PMA (30 ng/mL) and ionomycin (500 ng/mL) (Sigma) as positive controls; (ii) RPMI medium as negative control; (iii) peptide from E1b (VNIRNCCYI); (iv) coculture with CMT64-6 cells previously treated during 48 h with 300 u/mL of IFNγ (Genzyme Cytokine Research Products, Cambridge, MA) [39, 46]. Splenocytes were stimulated overnight, 37°C. Then, Brefeldin A was added to the wells to a final concentration of 10 μg/mL and incubated for 4 h, 37°C. After the plate was spun and vortexed, the cells were stained for surface antigens, using the antibodies anti-CD3, anti-CD8 previously mentioned. Cells were then incubated in fixation/permeabilization solution during 20 minutes at 4°C and washed twice in BD Perm/Wash buffer (using BD Cytofix/Cytoprem, BD Bioscience). The cells were stained for detection of the intracellular marker IFNγ using a monoclonal anti mouse IFNγ (clone XMG1.2, eBioscience) prepared in Perm/Wash buffer at 4 μg/ml, washed, acquired using the LSR II flow cytometer and analyzed by Flowjo analysis software.

### Cytokine array and quantification

mMSCs and mCelyvir were seeded in p60 Petri dishes. After 24 h, supernatants were collected and the protein pattern was analyzed using proteome profiler array kits. For analyzing the expression profile of angiogenesis-related proteins, we used the Proteome Profiler Mouse Angiogenesis Array Kit, and for analyzing the cytokine pattern, the Proteome Profiler Mouse Array Panel A kit. Both kits were used according to manufacturer's recommendation (R&D Systems, Minneapolis, MN).

### Enzyme-linked immunosorbent assay (ELISA)

mMSCs and mCelyvir were seeded at 4 × 10^4^ cells in 24-well plates in 1 mL medium containing 20% fetal bovine serum. At the indicated times, culture medium was collected and CXCL10 protein levels were measured by enzyme-linked immunosorbent assay (ELISA) using a commercial kit and following the manufacturer's instructions (Mouse CRG-2/IP-10 Ray Bio ELISA Kit, RayBiotech, Norcross, GA).

### *In vitro* cell migration assays

Transwells (8 μm pore filters, BD Biosciences) were coated with 0.1% gelatin (Sigma) and 5 × 10^4^ mMSCs/mCelyvir were transferred to the upper chambers. Cells were incubated in the presence of DMEM (negative control) or 2 × 10^5^ of cells (CMT64-6, RENCA or B16 (murine tumoral cell lines), or MEFs (normal fibroblast cells)) in the bottom chamber for 6 or 24 hours. A negative control assay was also performed by incubating the cells with DMEM alone in the bottom chamber. A positive control assay was also included, by incubating cells with DMEM supplemented with 5% FBS. Migrated cells were fixed and stained with crystal violet. For statistical analysis, cells were manually counted in 10 high-power fields (HPFs). Each experiment was repeated three times (*n* = 6). For splenocyte migration assay, splenocytes were analyzed as previously described [47].

### Statistical analysis

Data were graphed and analyzed using StataSE12 statistical analysis software (college Station, TX) and GraphPad Prism (GraphPad Software, La Jolla, CA). All experiments were performed in a blinded manner and repeated independently under identical conditions. Data distribution was examined first and transformations were applied as appropriate. Results were expressed as the mean ± SEM and were analyzed as follow: for continuous variables, comparisons between two groups was tested using 2-tailed Student's *t* test, and comparisons between more than two groups were examined using ANOVA with appropriate post-hoc test. To compare groups, ANOVA was performed, followed by Tukey–Kramer post hoc test if the *P value* was < 0.05. Post-hoc analyses were conducted using the Newman–Keuls method for multicomparison procedures. All statistical tests were two tailed. A confidence interval of 95% and *P value* < 0.05 was used to establish significance. Statistical significance was defined as *P < 0.05, **P < 0.01 and ***P < 0.001.

## SUPPLEMENTARY MATERIALS FIGURES AND TABLES


