# Supplementary material for: Mesenchymal stem cell carriers enhance antitumor efficacy of oncolytic adenoviruses in an immunocompetent mouse model
Source: Oncotarget. 2017 May 2;8(28):45415–31. doi: 10.18632/oncotarget.17557 (PMC5542197; doi:10.18632/oncotarget.17557)
Supplement: Supplementary file 1 [file oncotarget-08-45415-s001.pdf]

Mesenchymal stem cell carriers enhance antitumor efficacy of oncolytic adenoviruses in an immunocompetent mouse model

Supplementary Materials

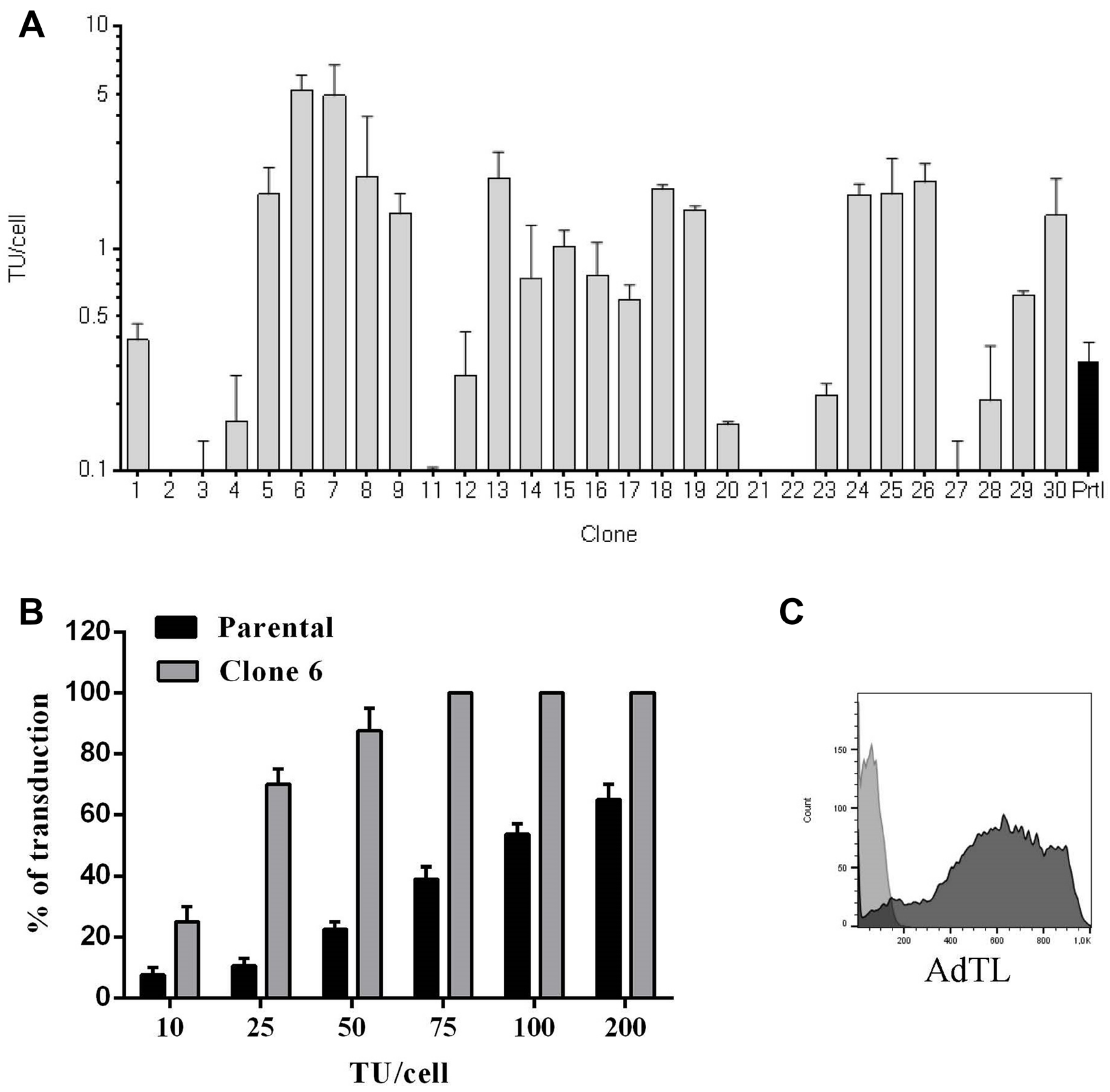

Supplementary Figure 1: Adenoviral replication in clones of the CMT64 cell line. (**A**) Monolayers of each clone from CMT64 cell line were isolated and infected. After 5 days, cell extracts were obtained and virus quantified by hexon-protein staining. (**B**) CMT64 parental and clone 6 cells were infected with AdTL at different MOI during 4 h and after 48 h the transduction percentage was analyzed by counting positive cells in a fluorescence microscopy. Bars represent mean of triplicates ± SEM. (**C**) mMSCs were transduced with AdTL and transduction percentage was detected by FACS.

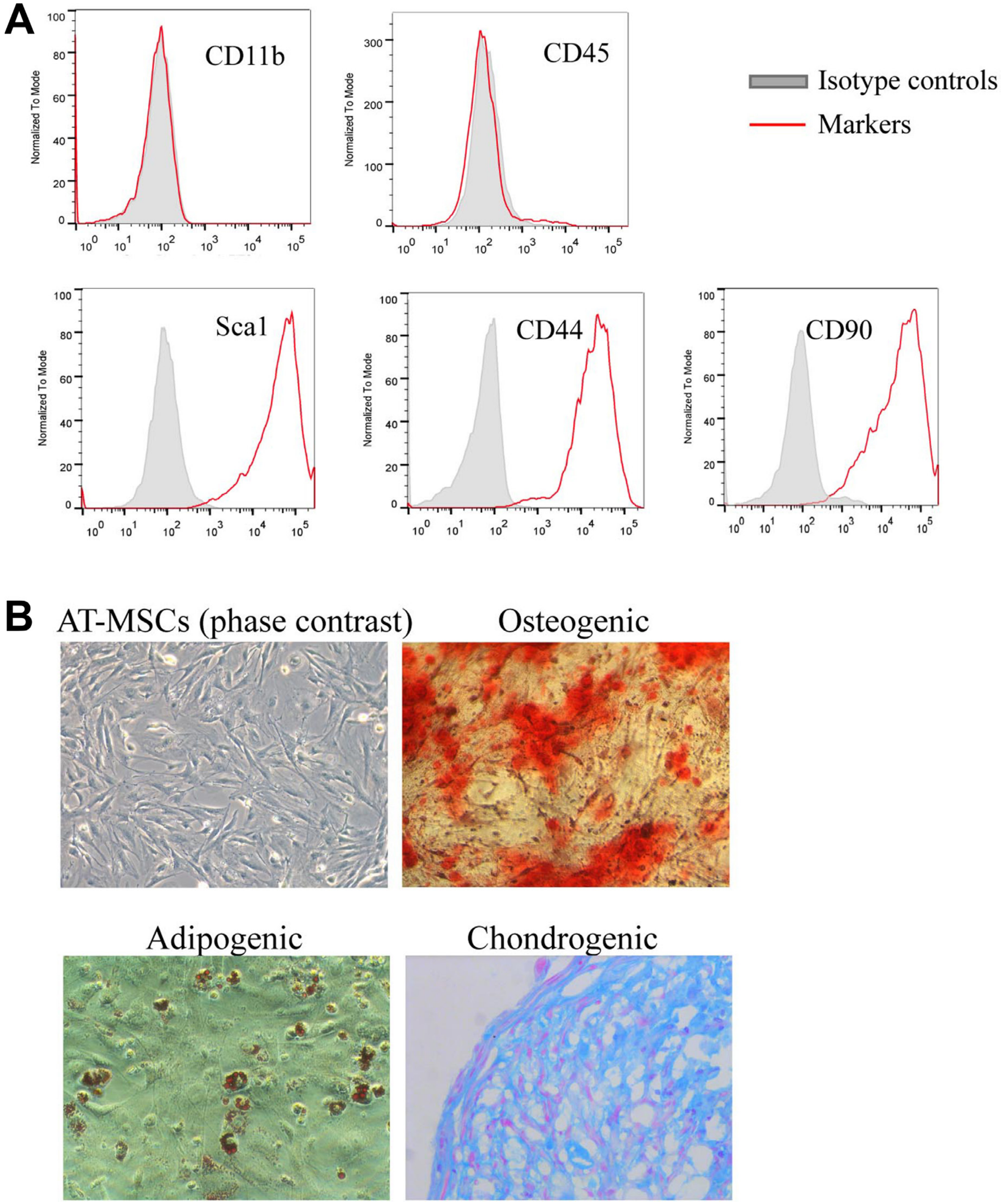

Supplementary Figure 2: Characterization of murine mesenchymal stem cells. (**A**) Cell surface marker expression was analyzed by flow cytometry. X-axes represent fluorescence intensity, and grey histograms are from isotype-matched controls. Sca1, CD44 and CD90 are considered as specific mMSC markers. (**B**) The cells produced calcified extracellular matrix upon culture in osteogenic differentiation medium as demonstrated by alizarin-red staining (positive staining for ALP activity) (upper right image); or fat deposits upon culture in adipogenic differentiation medium as shown by oil-red O staining of intracytoplasmatic lipid droplets (lower left image). A pellet culture system in the presence of TGF-β (1 pg/ml) was used for chondrogenic differentiation. Alcian blue staining revealed presence of collagen (blue), also counterstaining with fast red was performed (lower right image).

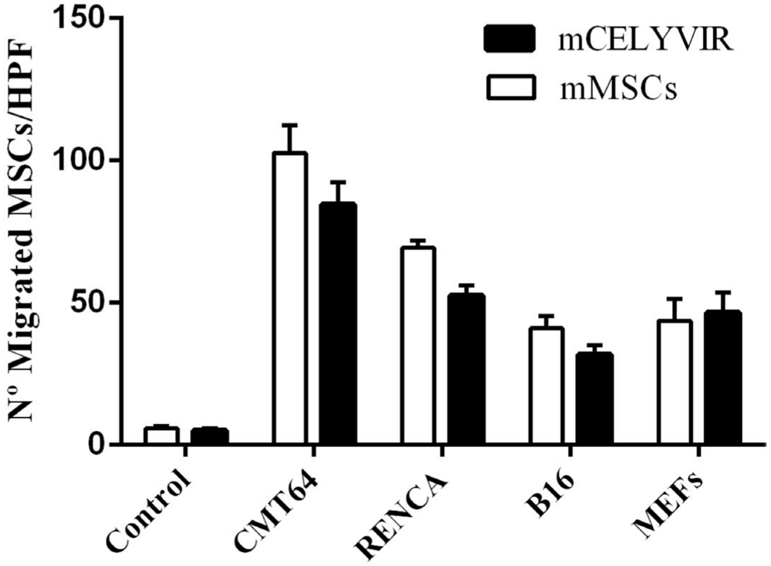

Supplementary Figure 3: Migration assay of mMSC/mCelyvir to different mouse cells. *In vitro* cell migration assay of mMSCs/mCelyvir against CMT64-6, RENCA, B16 or MEF cells was performed using 8 µm pore filters. mMSCs/mCelyvir were transferred to the upper chamber. Cells were incubated in presence of DMEM (as negative control) or the murine cells above mentioned in the bottom chamberS for 24 h. Migrated cells were fixed, stained with crystal violet and manually counted. The graph shows the average number of migrated mMSCs/mCelyvir cells in 10 HPF (high-power field). Bars represent mean of triplicates ± SEM.

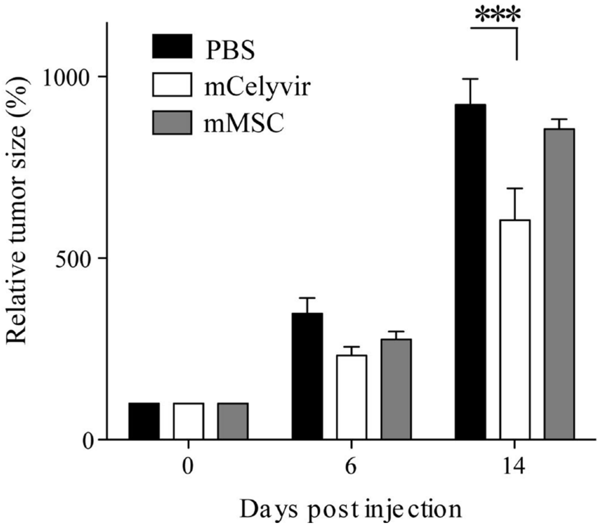

Groups	IP	IT
PBS	PBS	PBS
mCelyvir	mCelyvir	PBS
mMSC	mMSC	PBS

Supplementary Figure 4: Murine MSCs do not have an antitumoral effect on CMT64 tumors. Mice were subcutaneously inoculated with CMT64-6 cell line and treated each 4 days with either PBS, mCelyvir or mMSC, following the scheme of Figure 4A. Tumor volume was measured periodically with a caliper, and volume was calculated. ANOVA was performed and statistical significance was defined as ***$P < 0.001$.

A

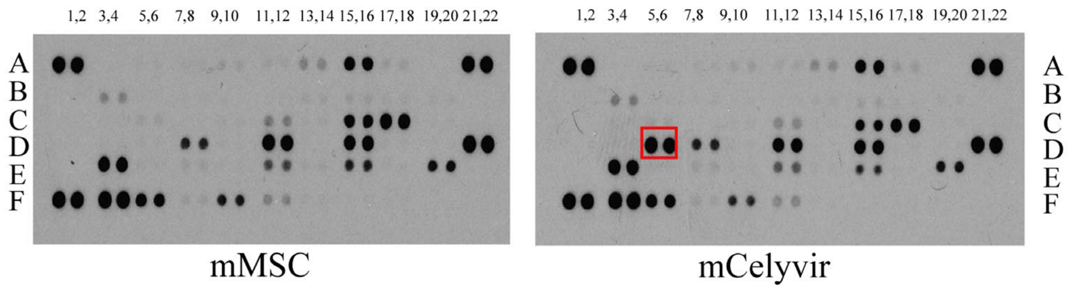

	1,2	3,4	5,6	7,8	9,10	11,12	13,14	15,16	17,18	19,20	21,22
A	Pos	-	ADAMTS1	Amphiregulin	Angiogenin	Angiopoietin-1	Angiopoietin-3	Coagulation F III	CXCL16	-	Pos
B	-	Cyr61	DLL4	DPPIV	DGF	Endoglin	Endostatin	IEndothelin-1	FGF acidic	FGF basic	-
C	-	KGF	Fractalkine	GM-CSF	HB-EGF	HGF	IGFBP-1	IGFBP-2	IGFBP-3	IL-1a	IL-1b
D	-	IL-10	CXCL10	KC	Leptin	MCP-1	MIP-1a	MMP-3	MMP-8	MMP-9	NOV
E	-	Osteopontin	PD-ECGF	PDGF-AA	PDGF-AB	Pentraxin-3	Platelet F 4	PIGF-2	Prolactin	Proliferin	-
F	Pos	SDF-1	Serpin E1	Serpin F1	Thrombospondin-2	TIMP-1	TIMP-4	VEGF	VEGF-B	Neg	-

B

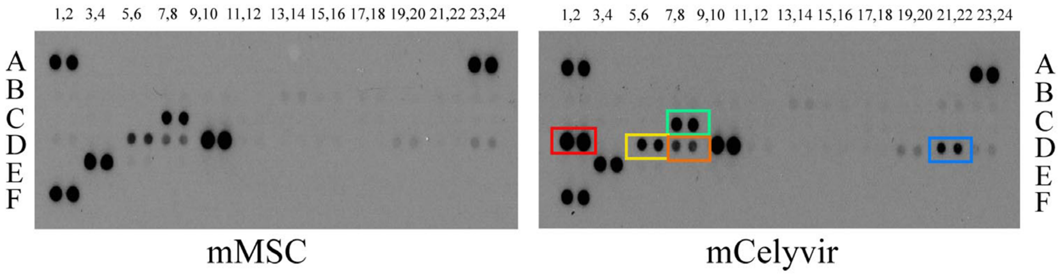

	**1,2**	**3,4**	**5,6**	**7,8**	**9,10**	**11,12**	**13,14**	**15,16**	**17,18**	**19,20**	**21,22**	**23,24**
**A**	Pos	-	-	-	-	-	-	-	-	-	-	Pos
**B**	BLC	C5a	G-CSF	GM-CSF	CCL1	CCL11	CD54	IFN-g	IL-1a	IL-1b	IL-1ra	IL-2
**C**	IL-3	IL-4	IL-5	IL-6	IL-7	IL-10	IL-13	IL-12p70	IL-16	IL-17	IL-23	IL-27
**D**	CXCL10	CXCL11	CXCL1	M-CSF	CCL2	CCL12	MIG	MIP-1a	MIP-1b	MIP-2	CCL5	CXCL12
**E**	CCL17	TIMP-1	TNF-a	TREM-1	-	-	-	-	-	-	-	-
**F**	Pos	-	-	-	-	-	-	-	-	-	-	Neg

Supplementary Figure 5: Angiogenesis and cytokine arrays. (**A**) Angiogenesis array: membrane with coordenates of all cytokines (upper panel) and table naming all cytokines (lower panel). (**B**) Cytokine array: Membranes with coordenates of all cytokines (upper panel) and table naming all cytokines (lower panel).

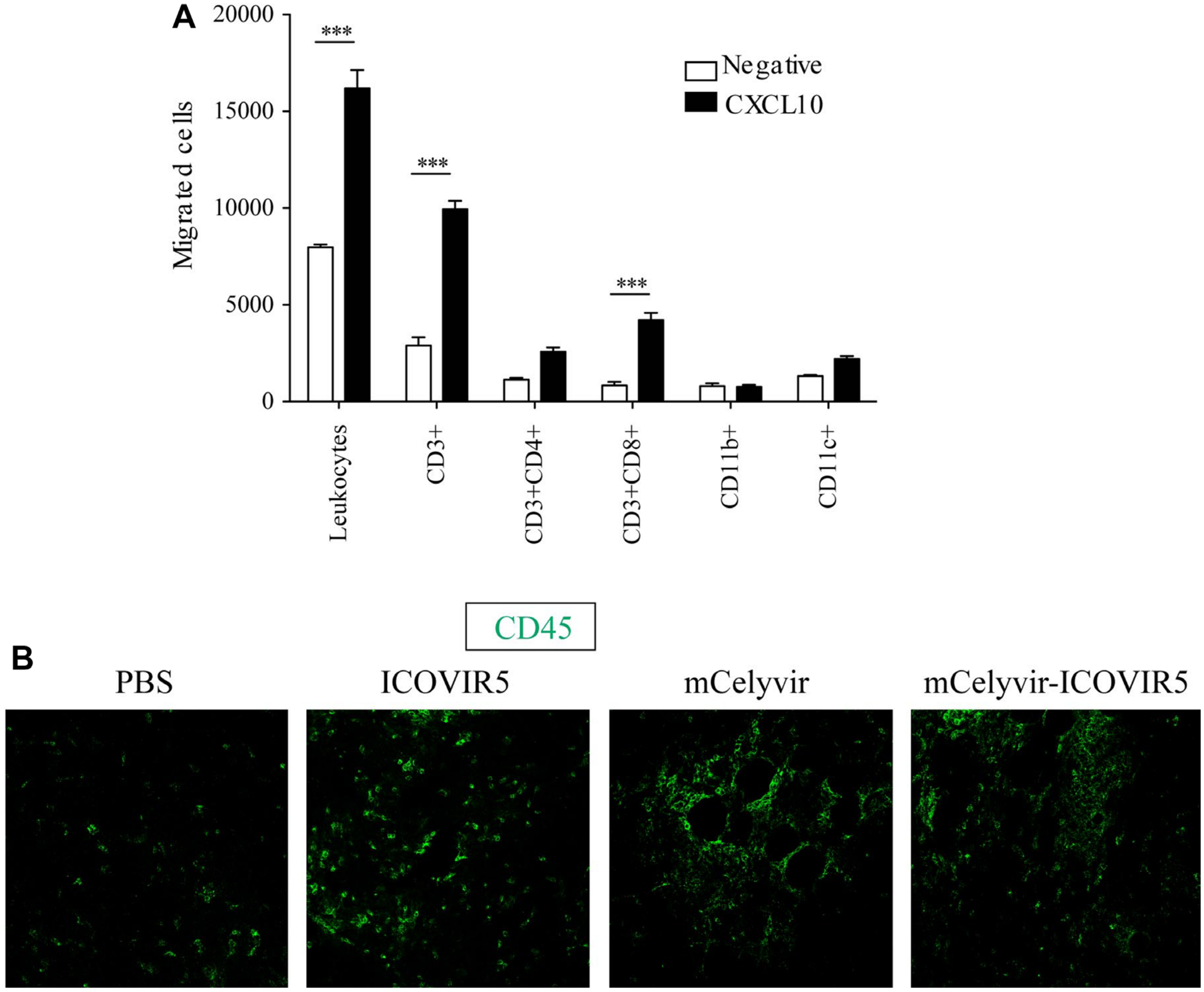

Supplementary Figure 6: Immune cell recruitment. (**A**) *In vitro* splenocyte cell migration assay towards CXCL10 was performed using transwells with 5 μm pore size. Splenocytes were transferred to the upper chambers. Cells were incubated in the presence of CXCL10 in the bottom chamber for 4 hours. Migrated cells were recovered, stained for different markers of immune subsets (CD45, CD3, CD4, CD8, CD11b, CD11c) and analyzed by flow cytometry. (**B**) Immunohistochemistry for the detection of immune system cells infiltrating the tumor was performed with an antibody to CD45 (green) on cryosections of tumors from each group of treatment. Student's t test was performed and statistical significance was defined as $^{***}P < 0.001$.

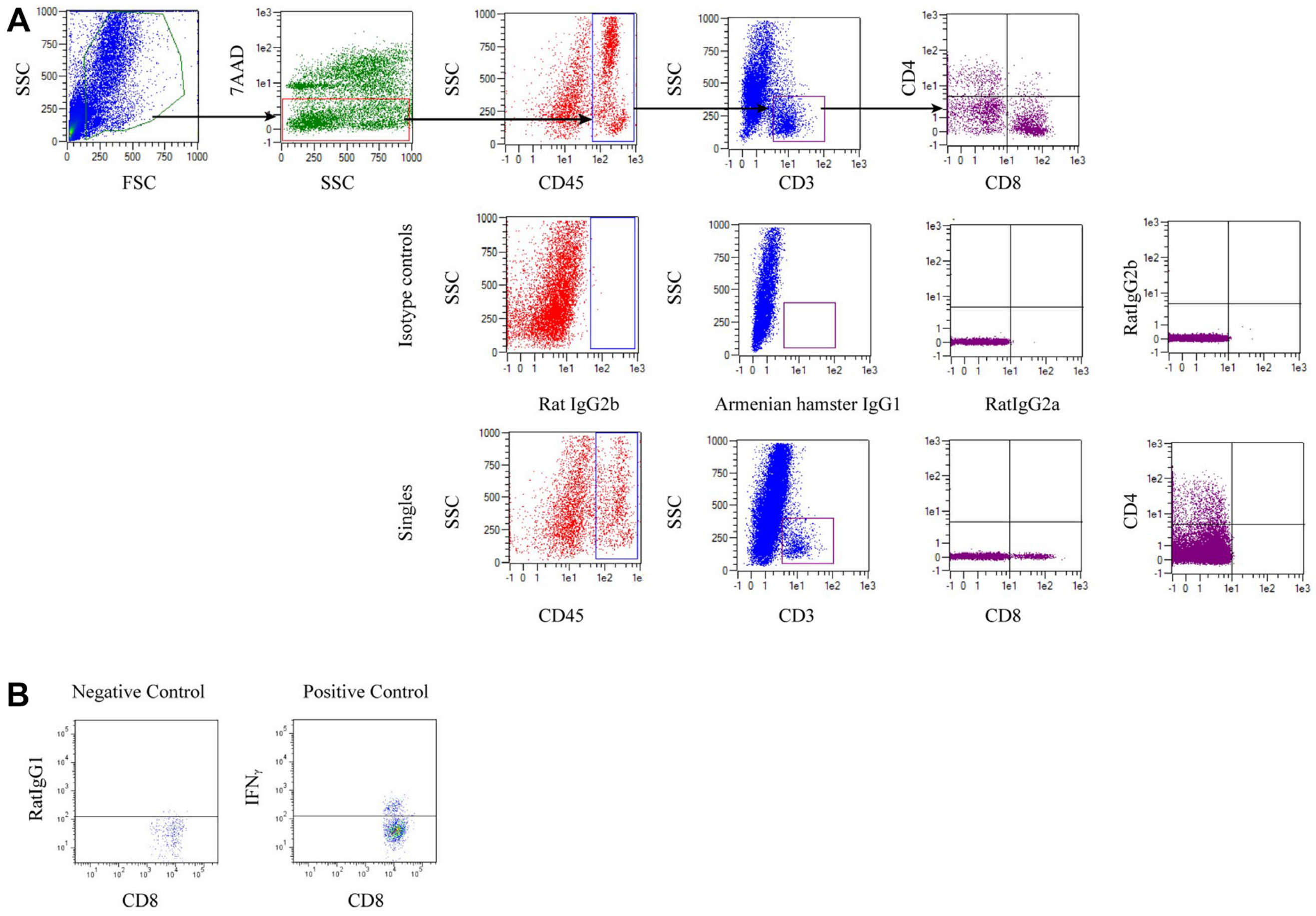

Supplementary Figure 7: Flow Cytometry gating strategy for immune infiltration. (**A**) For evaluation of tumor immune infiltration by FACS, CMT64-6 were disaggregated and cell suspensions were analyzed. We proceeded to the strategy showed on these representative dot plots. First, we made a gate on FSC-SSC, then excluded the necrotic cells by avoiding $7AAD^+$ events. Within this subset, we gated on $CD45^+$ cells and then within $CD3^+$ cells, where we analyzed the $CD4^+$/$CD8^+$ T cells (first row). We used Isotype controls for each antibody (second row) and individual staining with each antibody for cytometer settings adjustment (third row). (**B**) For the analysis of *ex vivo* IFNγ production in spleen-derived $CD8^+$ T cells obtained from treated mice, we performed the gating strategy as in Supplementary Figure 7A and used as positive control PMA + Ionomycin treated splenocytes. We show representative dot plots of this positive control and the isotype control used for IFNγ.
